# Comparative Benchmark of Seven Large Language Models for Traumatic Dental Injury Knowledge

**DOI:** 10.1055/s-0045-1812064

**Published:** 2025-10-22

**Authors:** Kittipat Termteerapornpimol, Sirinya Kulvitit, Sasiprapa Prommanee, Zohaib Khurshid, Thantrira Porntaveetus

**Affiliations:** 1 Department of Occlusion, Faculty of Dentistry, Chulalongkorn University, Bangkok, Thailand; 2 Department of Operative Dentistry, Faculty of Dentistry, Chulalongkorn University, Bangkok, Thailand; 3 Clinical Research Center, Faculty of Dentistry, Chulalongkorn University, Bangkok, Thailand; 4 Department of Prosthodontics and Dental Implantology, College of Dentistry, King Faisal University, Hofuf, Kingdom of Saudi Arabia; 5 Center of Excellence in Precision Medicine and Digital Health, Geriatric Dentistry and Special Patients Care International Program, Department of Physiology, Faculty of Dentistry, Chulalongkorn University, Bangkok, Thailand

**Keywords:** traumatic dental injuries, artificial intelligence, large language models, chatbots, DeepSeek, health care

## Abstract

**Objectives:**

Traumatic dental injuries (TDIs) are complex clinical conditions that require timely and accurate decision-making. With the rise of large language models (LLMs), there is growing interest in their potential to support dental management. This study evaluated the accuracy and consistency of DeepSeek R1's responses across all categories of TDIs and benchmarked its performance against other common LLMs.

**Materials and Methods:**

DeepSeek R1 and six other LLMs, ChatGPT-4o mini, ChatGPT-4o, Claude 3.5 Sonnet, Claude 3 Opus, Gemini 1.5 Flash, and Gemini 1.5 Advanced, were assessed using a validated question set (125 items) covering five subtopics: general introduction, fractures, luxations, avulsions of permanent teeth, and TDIs in the primary dentition (25 items per group) with a specific prompt. Each model was tested with five repetitions for all items.

**Statistical Analysis:**

Accuracy was calculated as the percentage of correct responses, while consistency was measured using Fleiss' kappa analysis. Kruskal–Wallis H and Dunn's post-hoc test were applied for comparisons of three or more independent groups.

**Results:**

DeepSeek R1 achieved the highest overall score of 86.4% ± 2.5%, despite the most inconsistent responses (κ = 0.694), statistically higher than those of ChatGPT-4o mini (74.7% ± 0.9%), Claude 3 Opus (75.2% ± 1.0%), and Gemini 1.5 Flash (73.85% ± 2.3%) ( *p* < 0.0001). Across all models, accuracy was notably lower for luxation injury questions (68.3% ± 3.2%).

**Conclusions:**

LLMs achieved moderate to high accuracy, yet this was tempered by varying degrees of inconsistency, particularly in the top-performing DeepSeek model. Difficulty with complex scenarios like luxation highlights current limitations in artificial intelligence (AI)'s diagnostic reasoning. AI should be viewed as a valuable dental educational and clinical adjunctive tool for knowledge acquisition and analysis, not a replacement for clinical expertise.

## Introduction

Artificial intelligence (AI) has been increasingly integrated across various dental specialties. 1 In endodontics, applications of AI in diagnosis, treatment planning, management, as well as surgical procedures have been introduced and explored by researchers and clinicians. 2 These include detection of periapical radiolucencies, 3 defining configurations of root and root canal system, 4 localization of apical foramen, and working length determination. 5 More recently, large language models (LLMs), basically known as chatbots such as ChatGPT, Gemini, and Claude, have caught widespread attention. Especially, the adoption of these AIs has increased among health care personnel. Recent surveys illustrated that roughly two-thirds of practicing physicians in the United States and one-third of academic dentists worldwide reported use of AI chatbots like ChatGPT. 6 7 The study also emphasized their potential in academic settings such as knowledge acquisition, patient education, research activities, and, interestingly, clinical decision-making. 6 Thus, demonstrating its potential to assist in solving clinical challenges in the endodontic field.

DeepSeek, a newly launched open-source LLM currently available as DeepSeek R1, might play an important role in health care setting as its performance was comparable or even superior to other proprietary LLMs. 8 Unlike other models, it is incorporated with a distinct feature, “Chain of thought” (CoT) reasoning, enabling the model to construct a step-by-step logical thinking prior to obtaining the final decision. 9 Mimicking the process of differential diagnosis typically made by the clinician, CoT may offer responses with human-like and well-supported reasons, particularly in complex clinical scenarios. As another fundamental component for clinical uses, DeepSeek is sometimes referred to as a “White Box” for its transparency, allowing users to access underlying thinking mechanisms, modifying existing algorithms to optimize its capability for specific tasks. 9

Notably, a previous study demonstrated that DeepSeek-3 achieved higher accuracy than ChatGPT-4o in generating differential diagnoses for oral lesions. Even without access to clinical and radiographic inputs, DeepSeek-3 produced differential diagnoses comparable to those of an oral medicine expert, outperforming ChatGPT-4o. 10 These findings suggest DeepSeek's considerable potential for application in the field of dentistry.

Despite well-established guidelines, traumatic dental injury (TDI) remains one of the topics in dentistry that most practitioners perceive themselves as inadequately knowledgeable. 11 12 According to Ozden et al, the accuracy of Google Bard and ChatGPT was 64 and 51%, respectively. 13 Another study included more models of LLM as well as their respective versions; the accuracy ranged from 46.7 to 76.7%, and ChatGPT 3.5 achieved the highest score. 14 Portilla et al subsequently identified the accuracy and consistency of Gemini, revealing its 80.8% accuracy with excellent consistency of 0.96. 15 However, a recent study evaluating ChatGPT-3.5's responses in the field of pediatric dentistry found that questions related to dental trauma yielded the lowest score, 16 highlighting the complex nature of dental trauma management that might challenge the capability of LLMs in providing qualified responses.

While recent studies have initiated the evaluation of LLMs in the context of TDIs, a comprehensive assessment of DeepSeek R1's diagnostic accuracy across all TDI classifications remains a critical gap. Establishing category-specific benchmarks is essential for advancing AI performance in dentistry. Therefore, this study aims to evaluate the diagnostic accuracy and response consistency of the DeepSeek R1 LLM across the full spectrum of TDI categories. Its performance will be benchmarked against other commonly used LLMs, and we hypothesize that all models will demonstrate significant variability in their accuracy and consistency for specific injury types.

## Materials and Methods

Following a previously conducted protocol, 13 dichotomous questions regarding TDIs and their answer keys were generated based on the 2020 International Association of Dental Traumatology (IADT) guidelines for the management of TDIs. 17 18 19 20 The question sets covered five subtopics of TDIs according to the guideline, general introduction, fractures, luxations, avulsions of permanent teeth, and TDIs in the primary dentition, with 25 items per group, some questions were adapted from Ozden et al. 13 The question sets were thoroughly reviewed and validated by an experienced endodontist with 11 years of experience (S.K.). The authors used the author's checklist for AI in dentistry from Schwendicke et al (available in Supplementary Table S1 , available in the online version only). 21

The validation confirmed that the questions and answer keys were accurate, logical, and evidence-based, following the 2020 IADT guidelines for the management of TDIs. We also confirmed that the questions fully covered all relevant TDI core knowledge, including clinical and radiographic assessment and findings, general management and patient instructions, endodontic treatment and consideration, follow-up regimens, favorable outcomes, and unfavorable outcomes, in accordance with the IADT guidelines. All questions can be accessed in Supplementary Table S2 , available in the online version only.

Seven LLMs were evaluated, including ChatGPT-4o mini and ChatGPT-4o (OpenAI, San Francisco, California, United States), Claude 3.5 Sonnet and Claude 3 Opus (Anthropic, San Francisco, California, United States), Gemini 1.5 Flash and Gemini 1.5 Advanced (Google LLC, Mountain View, California, United States), and DeepSeek R1 (DeepSeek, Hangzhou, China). Six LLMs were evaluated between August and December 2024, while DeepSeek R1 was assessed separately from January to March 2025 following its release. Specifically, each LLM was prompted with: “Please answer the following Yes/No question as a dentist…,” followed by the questions listed in Supplementary Table S2 , available in the online version only. An example of a full prompt is stated below, according to an item from the General introduction section. “Please answer the following Yes/No question as a dentist, Enamel infraction is the only TDI that requires no follow-up.” All responses were recorded in Microsoft Excel Version 16.95 (Microsoft, Redmond, Washington, United States) and subsequently scored by the research team (K.T. and S.P.). The dichotomous answers, along with the accompanying explanations, were again reviewed and verified by an experienced endodontist (S.K.). One mark was given for each correct dichotomous response, and no marks were given for incorrect dichotomous responses. Five repeated assessments were performed for each LLM using its default platform settings on Chrome for Windows Version 129.0.6628.3 (Google LLC, Mountain View, California, United States). To minimize bias, the chat history was manually cleared between questions, and separate accounts were utilized for each LLM. Additionally, the “Memory” feature was disabled in the personalization settings for ChatGPT-4o and ChatGPT-4o mini assessments.

To compare accuracy of LLMs, percentages of correct responses (%Accuracy) were calculated for overall and specific TDIs. Fleiss' kappa analysis (κ) was used to evaluate agreement of repeated binary “Yes” or “No” responses from each LLM based on an established metric, 22 ranging agreement from poor (κ < 0.2) to very good (κ = 0.80–1.00). Higher Fleiss' kappa values indicate greater consistency of responses, reflecting more reliable model performance. Descriptive statistics and Fleiss' kappa analysis were obtained from the IBM SPSS version 29.0.2.0 (IBM Corporation, Armonk, New York, United States). While other statistical analyses were performed using GraphPad Prism 10 (GraphPad Software Inc., San Diego, California, United States). Prior to analyses, the Shapiro–Wilk test was used to assess datasets' normality. One-way ANOVA (analysis of variance) was used for comparisons of normally distributed data, while Kruskal–Wallis H and Dunn's post-hoc test were applied for comparisons of three or more independent groups with skewed data. Effect size ( *η ^2^* ) was calculated following a previously described method. 23 A significance level less than 0.05 was considered statistically significant.

## Results

The overall scores showed that DeepSeek R1 achieved the highest accuracy among the tested LLMs (86.4% ± 2.5%, 95% confidence interval [CI]: 83.3–89.5), followed by ChatGPT-4o (78.6% ± 1.0%, 95% CI: 77.3–79.9) and Gemini 1.5 Advanced (77.9% ± 1.7%, 95% CI: 75.9–80.0). The accuracy of DeepSeek R1 was statistically higher than ChatGPT-4o mini, Claude 3 Opus, and a strongly significant difference when compared with Gemini 1.5 Flash, a basic model of the Gemini series. While all LLMs resulted in over 70% mean accuracies for total questions, Gemini 1.5 Flash presented the lowest accuracy with a relatively high standard deviation of 73.8% ± 2.3% (95% CI: 70.9–76.6; Fig. 1 ).

**Fig. 1:**
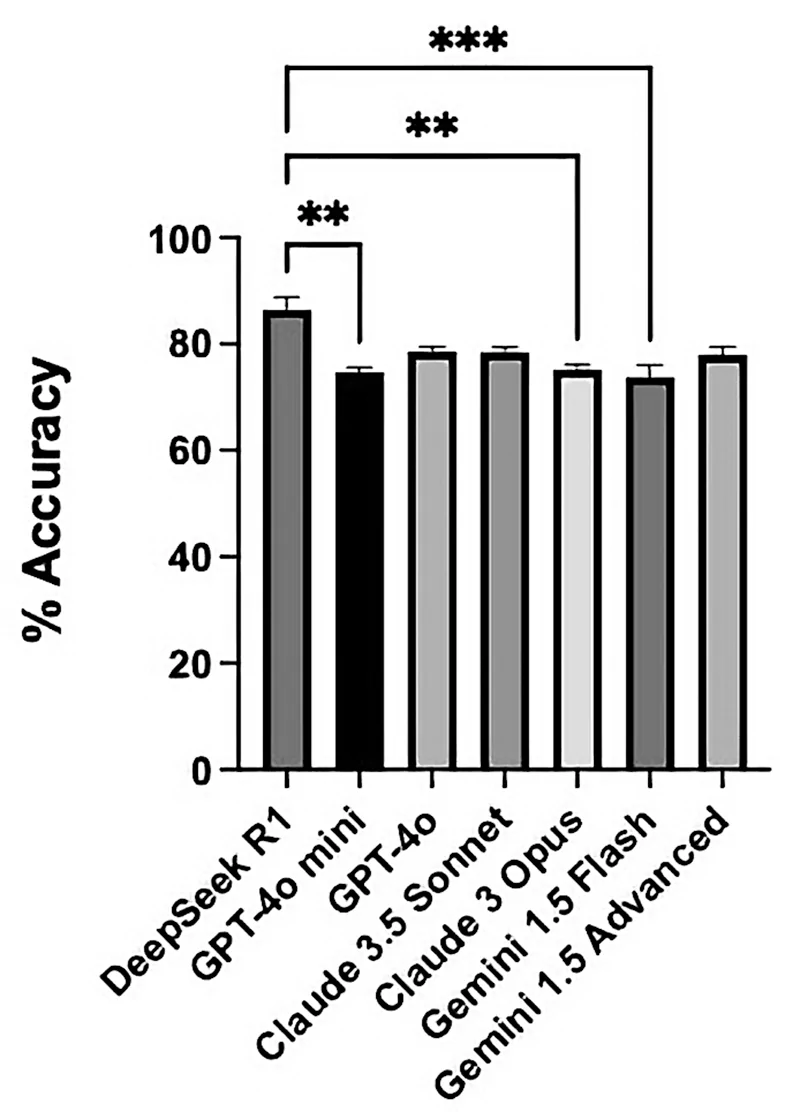
Overall accuracy of selected LLMs. Bars show the mean percentage of correct answers ± SD obtained from five independent runs per model. DeepSeek R1 achieved the highest accuracy (86.4% ± 2.5%; 95% CI: 83.3–89.5), significantly surpassing ChatGPT-4o mini, Claude 3 Opus (** *p* < 0.01), and Gemini 1.5 Flash (*** *p* < 0.001). ChatGPT-4o (78.6% ± 1.0%; 95% CI: 77.3–79.9) and Gemini 1.5 Advanced (77.9% ± 1.7%; 95% CI: 75.9–80.0) followed, while Gemini 1.5 Flash showed the lowest performance (73.8% ± 2.3%; 95% CI: 70.9–76.6). Statistical comparisons were performed with one-way ANOVA followed by Dunn's post-hoc testing; the symbol “**” indicates *p* < 0.01, *** indicates *p* < 0.001. All models exceeded the 70% accuracy threshold. CI, confidence interval; LLM, large language model; SD, standard deviation.

To specifically compare performances of each LLM, we made comparisons of accuracy of each LLM when asked about general introduction and specific types of TDIs according to 2020 IADT guidelines for the management of TDIs ( Table 1 , Fig. 2 ). Notably, DeepSeek R1 showed the highest accuracy on all topics except general introduction with varying degrees of statistical significance.

**Table 1:** Mean accuracy and standard deviation of each LLM across traumatic dental injury categories

	General introduction				Fractures				Luxations				Avulsion				TDI in the primary dentition				Total			
		95% CI				95% CI				95% CI				95% CI				95% CI				95% CI		
Models	Mean	LL	UL	SD	Mean	LL	UL	SD	Mean	LL	UL	SD	Mean	LL	UL	SD	Mean	LL	UL	SD	Mean	LL	UL	SD
**DeepSeek R1**	**91.2**	84.7	97.7	5.2	**92.0**	87.0	97.0	4.0	**79.2**	75.0	83.4	3.3	**87.2**	79.0	95.4	6.6	**82.4**	78.0	86.8	3.6	**86.4**	83.3	89.5	2.5
**GPT-4o mini**	**83.2**	81.0	85.4	1.8	**80.8**	76.6	85.0	3.3	**60.0**	60.0	60.0	0.0	**75.2**	73.0	77.4	1.8	**74.4**	71.7	77.1	2.2	**74.7**	73.6	75.9	0.9
**GPT-4o**	**92.0**	92.0	92.0	0.0	**84.0**	79.0	89.0	4.0	**70.4**	67.7	73.1	2.2	**72.0**	72.0	72.0	0.0	**74.4**	71.7	77.1	2.2	**78.6**	77.3	79.9	1.0
**Claude Sonnet**	**72.0**	64.2	79.9	6.3	**88.0**	81.9	94.1	4.9	**70.4**	64.7	76.1	4.6	**80.0**	75.0	85.0	4.0	**81.6**	78.9	84.3	2.2	**78.4**	77.2	79.6	1.0
**Claude Opus**	**84.0**	84.0	84.0	0.0	**84.8**	82.6	87.0	1.8	**60.8**	58.6	63.0	1.8	**70.4**	67.7	73.1	2.2	**76.0**	76.0	76.0	0.0	**75.2**	74.0	76.4	1.0
**Gemini 1.5 F**	**84.8**	76.7	93.0	6.6	**82.4**	79.7	85.1	2.2	**62.4**	56.7	68.1	4.6	**70.4**	62.9	77.9	6.1	**68.8**	64.6	73.0	3.3	**73.8**	70.9	76.6	2.3
**Gemini 1.5 Adv**	**89.6**	86.9	92.3	2.2	**84.0**	84.0	84.0	0.0	**74.4**	67.7	81.1	5.4	**71.2**	69.0	73.4	1.8	**70.4**	66.0	74.8	3.6	**77.9**	75.9	80.0	1.7

Abbreviations: CI, confidence interval; Claude Opus, Claude 3 Opus; Claude Sonnet, Claude 3.5 Sonnet; Gemini 1.5 Adv, Gemini 1.5 Advanced; Gemini 1.5 F, Gemini 1.5 Flash; GPT-4o mini, ChatGPT-4o mini; GPT-4o, ChatGPT-4o; LL, lower limit; SD, standard deviation; TDI, traumatic dental injury; UL, upper limit.

Note: Statistical significance was evaluated using Kruskal–Wallis tests. The following *p* -values were obtained for each category: general introduction: *p* = 0.0003, *η ^2^* = 0.68; fractures: *p* = 0.0053, *η ^2^* =0.44; luxations: *p* < 0.0001, *η ^2^* = 0.81; avulsion: *p* = 0.0003, *η ^2^* = 0.68; primary dentition: *p* < 0.0001, *η ^2^* = 0.81; total: *p* < 0.0001, *η ^2^* = 0.81. *p* <0.05 indicates a statistically significant difference in LLM accuracy across the models within that category.

**Fig. 2:**
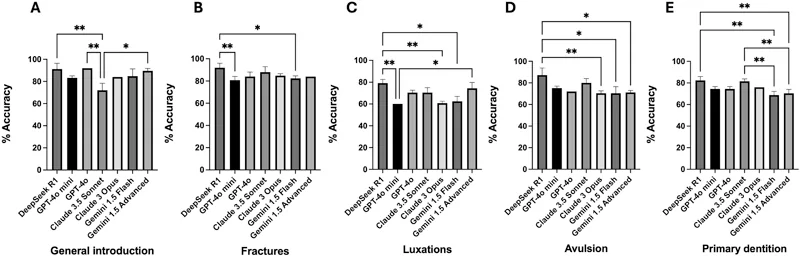
Mean accuracy and standard deviation of each LLM across traumatic dental injury categories. Bars show mean %accuracy ± SD across five topics. DeepSeek R1 consistently ranked highest, especially in luxation and avulsion. Claude 3.5 Sonnet showed top accuracy in primary dentition. ( **A** ) General introduction. ( **B** ) Fractures. ( **C** ) Luxations. ( **D** ) Avulsion. ( **E** ) TDI in the primary dentition. The symbol “*” indicates statistically significant difference ( *p* < 0.05), and ** indicates *p* < 0.01. LLM, large language model; SD, standard deviation; TDI, traumatic dental injury.

Considering accuracy of responses on general introduction, the three highest accuracies were obtained from ChatGPT-4o (92% ± 0.0%, 95% CI: 92.0–92.0), DeepSeek R1 (91.2% ± 5.2%, 95% CI: 84.7–97.7), and Gemini 1.5 Advanced (89.6% ± 2.2%, 95% CI: 86.9–92.3), which are significantly greater than Claude 3.5 Sonnet (72.0% ± 6.3%, 95% CI: 64.2–79.9).

Regarding the fracture injuries, most LLMs showed comparable accuracies on questions except ChatGPT-4o mini (84.0% ± 4.0%, 95% CI: 79.0–88.9) and Gemini 1.5 Flash (82.4% ± 2.2%, 95% CI: 79.7–85.1), which obtained significantly lower accuracies compared with DeepSeek R1 (92.0% ± 4.0%, 95% CI: 87.0–97.0).

The higher accuracy of DeepSeek R1 compared with other LLMs was more pronounced statistically when considering questions regarding luxation and avulsion injuries, as shown in Fig. 2(C, D) . Lastly, regarding TDIs in primary dentition, Claude 3.5 Sonnet outperformed other models, with statistical significance when compared with Gemini 1.5 Flash and Gemini 1.5 Advanced, as well as DeepSeek R1.

Considering the accuracy of responses across each category of TDIs, the fractures and general introduction categories received the highest scores, both significantly higher than those for luxations, avulsion, and injuries in the primary dentition. Among all categories, the luxations category had the lowest accuracy score, which was significantly lower than the scores for fractures, general introduction, and injuries in the primary dentition ( Supplementary Table S3 , available in the online version only).

All models consistently showed good to very good agreement (κ = 0.694–0.924) according to the Fleiss' kappa analysis ( Table 2 ). Specifically, ChatGPT-4o mini delivered outstandingly consistent responses with κ of 0.924, followed by ChatGPT-4o (0.886), Claude 3 Opus (0.871), and Gemini 1.5 Advanced (0.847), which corresponded to very good agreement. The model with the lowest consistency was DeepSeek R1 (0.694), followed by Gemini 1.5 Flash (0.698) and Claude 3.5 Sonnet (0.773). It is noteworthy that despite the best performance in overall accuracy, DeepSeek R1 exhibited the lowest consistency with κ of 0.694.

**Table 2:** Consistency of each LLM based on Fleiss' kappa analysis

	Fleiss' kappa reliability
**DeepSeek R1**	0.694
**ChatGPT-4o mini**	0.924
**ChatGPT-4o**	0.886
**Claude 3.5 Sonnet**	0.773
**Claude 3 Opus**	0.871
**Gemini 1.5 Flash**	0.698
**Gemini 1.5 Advanced**	0.847

Abbreviation: LLM, large language model.

Note: Consistency of seven LLMs in evaluating traumatic dental injury responses. ChatGPT-4o mini demonstrated the highest consistency (κ = 0.924), followed by ChatGPT-4o (κ = 0.886) and Claude 3 Opus (κ = 0.871). The lowest consistency was observed in DeepSeek R1 (κ = 0.694) and Gemini 1.5 Flash (κ = 0.698). Higher Fleiss' kappa values indicate greater agreement among raters, reflecting more reliable model performance.

## Discussion

The overall accuracy in this study ranged from 73.8 to 86.4%, with DeepSeek R1 achieving a significantly higher overall accuracy than the other evaluated models, and Gemini 1.5 Flash demonstrating the lowest performance in accuracy. Therefore, the null hypothesis of no difference in accuracy among LLMs was rejected. Notably, although DeepSeek R1 was recorded as the highest accuracy, its consistency was rated at a “good” level rather than “very good.” The accuracy recorded in this study is higher than that reported in a previous study on the same topic using ChatGPT-3.5 and Gemini, where the correct response rate was only 57.5%. 13 This discrepancy may be attributed to differences in prompt design. Specifically, the earlier study did not instruct the AI to respond as a dentist, whereas the present study employed a targeted prompt. Another study utilizing a similar prompt format reported a comparable accuracy level of 80.8% when using the Gemini model. 15

The comparative performance of specific LLMs in answering dental trauma questions remains inconclusive. In our study, DeepSeek R1 achieved the highest accuracy (86.4% ± 2.5%), followed by ChatGPT-4o (78.6% ± 1.0%) and Gemini 1.5 Advanced (77.9% ± 1.7%). Similarly, Mustuloğlu and Deniz reported that ChatGPT-4.0 (95.6%) outperformed Gemini (78.3%) in responding to questions on the emergency management of avulsion injuries. 24 In contrast, Ozden et al found that Gemini achieved a higher correct-answer rate (64.0%) than ChatGPT-3.5 (51.0%), and Tokgöz Kaplan et al reported that Gemini 1.5 Pro scored significantly higher for dental avulsion knowledge (4.2% ± 0.9) compared with ChatGPT-3.5 (3.5% ± 1.4). 25 Collectively, the current evidence remains insufficient to confirm the superiority of any single LLM for this specific task; however, their potential as adjunct clinical or educational tools is evident.

The consistency of LLMs is highly variable across models, tasks, and assessment methods. For instance, in dental trauma and endodontic knowledge, ChatGPT (version 3.5) demonstrated agreement values ranging from as low as 0.266 13 to as high as 0.987. 14 Similarly, while Gemini has previously achieved excellent agreement in dental trauma tasks, 14 15 26 our study observed lower consistency in the Gemini 1.5 Flash model. DeepSeek R1, which showed the lowest consistency in our evaluation (κ = 0.694), was also previously reported with moderate agreement ( *r* = 0.615) in pediatric dentistry tasks. 27 These discrepancies reflect the dynamic and evolving nature of LLM performance, as model updates are incessantly released. Importantly, the variability in consistency underscores the need for caution in relying on LLM-generated responses, particularly in high-stakes clinical scenarios, as it carries a risk of critical errors.

When comparing AI models from the same provider, the paid version of ChatGPT-4o demonstrated higher accuracy than the public version. Similarly, the paid version of Gemini 1.5 Advanced outperformed the free version, in both accuracy and consistency. However, these trends did not reach statistical significance. Besides, all paid AI models achieved a level of consistency classified as “very good.” In comparison with previous research, the reliability of the Gemini 1.5 Flash model in the present study was rated as good (κ = 0.698), which is lower than the excellent reliability ( *r* = 0.952) reported in an earlier study. 14

Focusing on different TDI categories, the lowest accuracy was observed in the luxation category, reflecting the greater complexity involved in diagnosing and managing such injuries. This limitation illustrates a broader concern regarding the current capabilities of LLMs in complex clinical scenarios. Despite their ability to rapidly arrange, integrate, and process vast amounts of information, thereby compensating for the limitations of human memory, 28 29 LLMs still lack the critical thinking and deep conceptual understanding required for health care decision-making. 30 This underscores the essential role of experienced clinicians, whose diagnostic reasoning and nuanced understanding remain irreplaceable in complex cases. While one study reported that LLMs performed comparably to, or even better than, clinicians in a written periodontology examination, 31 another study suggested contrasting results, 32 reflecting the variability in LLM performance across different contexts. These findings highlight the complementary nature of LLMs and clinicians, where each may help offset the other's weaknesses.

Our study reinforces the findings of previous research, 13 14 15 highlighting the imperfect accuracy and consistency of LLMs in TDI knowledge. Consequently, their application in clinical decision-making should be limited to experienced dental personnel who are capable of critically interpreting AI-generated responses. Knowledgeable users combined with appropriate AI use strategies are essential to maximize AI capabilities in real-world settings. Reliable and updated data sources, such as clinical guidelines, scientific papers, or official statements, selectively provided by users are a prerequisite to obtain high-quality outputs. 33 Furthermore, prompts should instruct the AI to adhere to the provided validated information, thereby minimizing the risk of hallucinations or algorithmic bias caused by the retrieval of inaccurate or misleading content. 28 34

The superior accuracy of DeepSeek R1 observed in our study is consistent with previous studies where DeepSeek's intriguingly high diagnostic accuracy in oral lesions of 91.6% was recorded, higher than ChatGPT-4o and the journal's readers. 35 This was also supported by another study showing its acceptable accuracy in diagnosing various oral pathology problems. 36 However, one common limitation of previous studies was that they did not either incorporate effective prompting strategies or declare their prompts when conducting the experiments. While there are many prompting strategies in LLMs from Zero-shot, CoT, to role-playing, the role assignment strategy has been demonstrated to be an effective method to enhance the reasoning capabilities of ChatGPT. 37

Although our study utilized a text-based approach to assess LLMs' capability, some LLMs allow incorporation of images into the prompt in addition to a textual description. Adding useful information, such as a clinical photograph or a radiographic image, into the prompt could also potentially enhance the accuracy of ChatGPT for the diagnosis of oral lesions. 10 35 The synergistic effects of such multimodal prompts on diagnostic accuracy in endodontics and dental trauma have yet to be systematically investigated.

A major strength of this study is the inclusion of a wide range of AI models, encompassing both free and paid versions. The questionnaire covered all categories outlined in the IADT guidelines, including general introduction, fractures and luxations, avulsion of permanent teeth, and injuries in the primary dentition. 17 18 19 20 Questions were based on the IADT guidelines and were presented using a standardized prompt. This approach was intended to elevate the quality of responses to a professional dental standard.

Closed-ended questions in a dichotomous format were utilized to ensure precise accuracy evaluation in this study, in accordance with previous studies. 13 15 24 Earlier research employed open-ended questions, assessed using the Global Quality Scale (GQS), and followed by evidence-based discussion until consensus was reached to determine accuracy. 38 39 Despite its simplicity and comprehensiveness, GQS is a subjective tool that depends on evaluators. In the context of accuracy assessment, closed-ended question eliminates GQS' drawbacks, as well as providing reliable and consistent objective assessment across different LLMs.

A key limitation of LLMs that should be taken into account while interpreting this study is the discrepancy between the dichotomous answers and the accompanying explanations. This inconsistency may be attributed to two types of error. First, inaccuracies within the explanations themselves, despite a seemingly correct dichotomous answer, may reflect factual errors commonly referred to as “hallucinations” in AI-generated content. For example, when DeepSeek R1 was asked a question regarding root fractures: “The apical part of a fractured root usually undergoes necrosis, leading to the need for root canal treatment.” Despite the correct “No” answer, looking carefully at its explanation revealed a consistent-looking misleading rationale, stating that “…necrosis typically involves the entire pulp rather than just the apical part…,” contradicting the fact that the coronal part of a fractured root is the part that usually undergoes necrosis, not the apical part 20 ( Supplementary Fig. S1A , available in the online version only). The number of this type of inaccuracy was recorded at 15 responses out of 4,375 total responses (0.3%). It was not only more frequent than another type of discrepancy resulting from complex prompts, but, crucially, it could also misguide clinical judgement and lead to harmful consequences. 30 As AI models have the capacity for machine learning based on user input, hallucinations could also be reduced by providing established standard guidelines as input and prompting the AI to respond specifically based on specific information. This approach is particularly applicable in areas where well-defined clinical guidelines exist, such as the management of dental trauma and infective endocarditis. 40 Second, complex prompts containing multiple sentences may lead the AI to address only part of the question, neglecting the rest of it. This results in an inaccurate dichotomous answer despite a totally correct underlying explanation ( Supplementary Fig. S1B , available in the online version only). Therefore, it is important to interpret both the dichotomous answer and the explanation together. Using more specific and straightforward questions may help reduce such discrepancies as well.

Future studies should aim to compare the accuracy and consistency of AI models with varying levels of expertise, clarifying the current standard of practice compared with practitioners, as well as exploring potential synergies when clinicians use AI as an adjunct tool, compared with unaided clinical judgment. Essentially, investigations on different prompting strategies are strongly encouraged to suggest an effective instruction of LLM uses in the field of dental trauma. With the advancing potential of LLMs such as DeepSeek in medical fields, longitudinal studies assessing the real-world application of this AI's accuracy, consistency, and practicality over time would serve as robust evidence for initiating its complete integration into daily practices.

## Conclusion

Our findings reveal a critical dichotomy in the current state of LLMs for dental trauma diagnostics. While models achieve moderate to high accuracy (73.8–86.4%), their utility is undermined by significant response inconsistency, a limitation particularly evident in their difficulty with complex scenarios like luxations, which points to deficits in nuanced diagnostic reasoning. Consequently, their immediate application is not as autonomous diagnostic tools, but rather as powerful educational aids for knowledge acquisition and analysis. Ultimately, this research provides foundational pilot data that frame current LLMs as a premature but promising technology, establishing a crucial benchmark for the future development of reliable, dentist-in-the-loop decision-support systems.
