# Supplementary material for: Comparative Benchmark of Seven Large Language Models for Traumatic Dental Injury Knowledge
Source: Eur J Dent. 2025 Oct 22;20(4):1215–22. doi: 10.1055/s-0045-1812064 (PMC13623463; doi:10.1055/s-0045-1812064)
Supplement: Supplementary file 1 [file 10-1055-s-0045-1812064-s2574427.pdf]

Supplementary Data

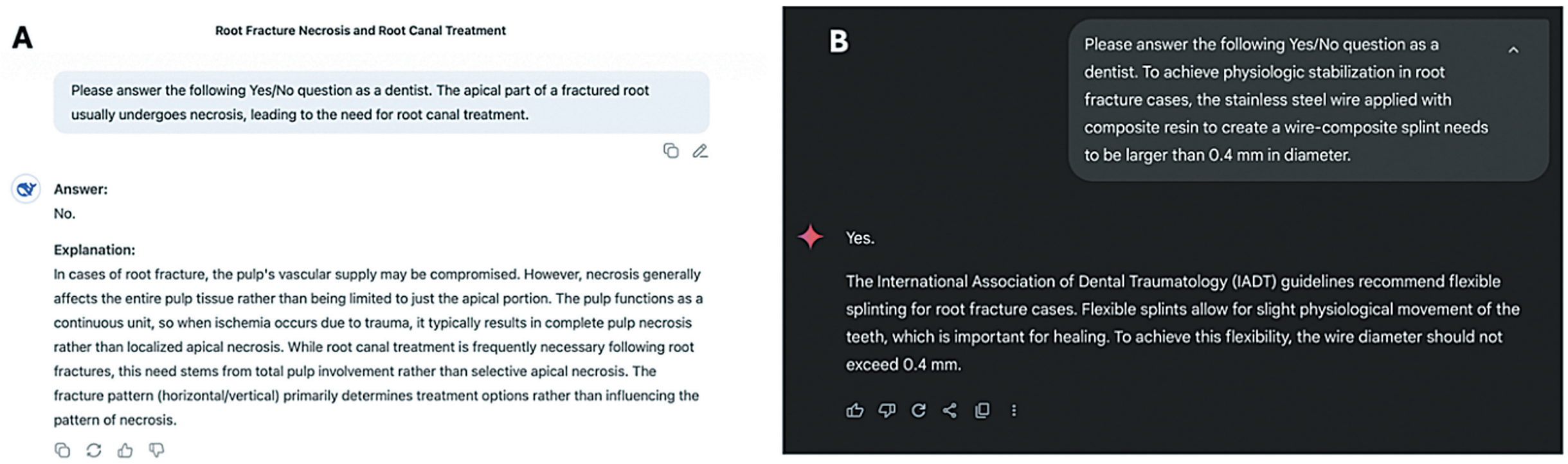

Supplementary Fig. S1 Examples of answer–explanation discrepancy generated by large language models. (**A**) DeepSeek R1. (**B**) Gemini 1.5 Advanced.

Supplementary Table S1 Checklist for study planning, conducting, and reporting

No.	Criteria	Fulfillment
1	Study goal (Consider relevance, scope and meaning, and limitations of the AI application.)	Yes
2	Study focus (Clarify if developing a new or validating an existing model, or if scope diagnostics or prognostics, etc.)	Yes
3	Data (Scrutinize the available dataset and mitigate bias, ensuring generalizability.)	Yes
4	Study aim (Define if the study is exploratory or hypothesis testing and consider implications for study conception.)	Yes
5	Reference test (Decide on a justifiable basis for a method to establish a reference test, especially when involving multiple annotators.)	Yes
6	Clustering (Consider clustering of teeth and patients in your dataset, e.g., during data partitioning to avoid data snooping bias.)	N/A
7	Test dataset (Report test metrics from an independent test dataset.)	Yes
8	Computational resources (Consider resources when working with larger datasets or complex models.)	N/A
9	Comparators (Compare your model against relevant comparators—experts, other models—using meaningful metrics.)	Yes
10	Title (Define that AI was used, and specify which one and for which focus and problem.)	Yes
11	Abstract (Present a structured summary of the study's aim, methods, results, and conclusion.)	Yes
12	Introduction (Sum up the clinical background, need for AI, achievements, limitations, goal, and hypothesis.)	Yes
13	Study design (Provide overview of study goal, data, modeling techniques, evaluation, and scope.)	Yes
14	Data (Source of data, inclusion/exclusion criteria, sampling, heterogeneity, partitioning, and access.)	Yes
14.1	Sampling (Details on image/data type and quality, inclusion/exclusion criteria, heterogeneity.)	Yes
14.2	Data protection (How data protection requirements were fulfilled.)	N/A
14.3	Missing data (Explain how missing data was handled.)	N/A
14.4	Data processing (Describe how data were extracted, transposed, preprocessed, etc.)	Yes
15	Reference test (Explain generation method, case definition, grading, thresholds, label unification.)	Yes
16	Sample size (If hypothesis testing, describe how the sample size was determined.)	Yes
17	Model (Provide information on model structure, software, hardware, etc.)	Yes
17.1	Model parameters (Describe model parameter initialization.)	Yes
18	Training (Describe training, data augmentation, hyperparameters, optimization, learning rate, batch size, etc.)	N/A
19	Justify the best-performing model (Describe model selection method and evaluation against hold-out set.)	N/A
20	Evaluation (Describe primary outcome and relevant metrics.)	Yes

(*Continued*)

 © 2025. The Author(s).

Supplementary Table S1 (*Continued*)

No.	Criteria	Fulfillment
21	Uncertainty (Describe how uncertainty is handled and presented.)	Yes
22	Explainability (Lay out how explainability and transparency were assessed.)	Yes
23	Results (Data flow, inclusion/exclusion, training/validation/test partitioning, dataset characteristics.)	Yes
23.1	Performance metrics and data partitions (Final test performance, benchmark comparisons, uncertainty estimates, explanation of incorrect predictions.)	Yes
24	Discussion (Summary, strengths/limitations, findings, implications, future directions.)	Yes
25	Other (Authorship, registration, study protocol, conflicts of interest.)	Yes

Supplementary Table S2 TDI question set by subtopic

General information	
1	Enamel infraction is the only traumatic dental injury that requires no follow-up.
2	The risk of severe pulp injury is increased when lateral luxation occurs simultaneously with crown fractures.
3	After fracture or luxation injuries, a mouthrinse containing alcohol is recommended for the patient's post-injury care due to its antibacterial effect.
4	The splint applied in trauma cases should be rigid.
5	Revascularization is more likely to occur in traumatized immature permanent teeth compared with mature permanent teeth.
6	Pulp canal obliteration is more likely to develop in traumatized immature teeth compared with fully developed teeth.
7	Root length and width should be evaluated at the follow-up visits for traumatized immature permanent teeth.
8	At the follow-up appointment, clinical examination of permanent dentition after traumatic dental injuries must always be accompanied by radiographic evaluation.
9	Rubber dam placement should always be attempted if root canal treatment is to be initiated in traumatized teeth. To avoid further trauma to the affected tooth, dentists can alternatively apply rubber dam clamps to neighboring teeth rather than the affected tooth itself.
10	Yearly follow-up for at least 5 years is recommended in permanent teeth with a history of extrusion, lateral luxation, intrusion, and avulsion.
11	A stainless steel wire up to 0.8 mm in diameter is typically suggested to create physiological stabilization of the luxated teeth or mobile segments.
12	The lack of responses to the pulp sensibility test alone is not conclusive for the diagnosis of pulp necrosis in traumatized teeth. However, the initial test result provides a baseline for further comparisons at follow-up visits.
13	Preventive endodontic treatment is reasonable for severely injured mature permanent teeth experiencing multiple traumas concurrently.
14	Splinting is usually needed in dislocated permanent teeth, while there are limited uses of splints in primary dentition.
15	Luxation injuries are the majority of injuries in primary dentition, while crown fractures are more common in permanent dentition.
16	Infraocclusion is an injury-specific outcome that should be considered for injuries involving damages to the periodontal ligament in permanent teeth.
17	Systemic antibiotics are always prescribed for traumatic dental injuries.
18	Root canal treatment should be initiated as soon as possible when there is a sign of inflammatory external root resorption after traumatic dental injuries.
19	The preferred intracanal medicament for treatment of inflammatory root resorption is triple antibiotic paste.
20	In cases of inflammatory external root resorption, calcium hydroxide should be placed for 3 weeks as an intracanal medicament and replaced every 3 months. Root canal obturation should be delayed until the healing sign is exhibited in the radiograph.
21	Preservation of pulp vitality using various techniques, such as partial pulpotomy, should be attempted in traumatized immature permanent teeth with viable pulp to allow further development of the affected teeth.
22	If decoronation of a replanted tooth is expected, dentists should carefully consider use of intracanal materials and placement duration.

 © 2025. The Author(s).

Supplementary Table S2 (*Continued*)

General information	
23	Repositioning and splinting are usually recommended for the displaced traumatized tooth. After splinting, any possible occlusal interferences should be eliminated.
24	A high metallic sound on percussion is a sign of ankylosis.
25	Unlike permanent teeth, traumatic dental injuries to primary teeth without any clinical signs or symptoms do not usually require radiographic examination, except those with an alveolar bone fracture.
Fractures	
1	Root canal treatment is the only treatment option for complicated crown fractures in teeth with complete root development.
2	To prevent further complications such as inflammatory root resorption after fracture injuries, root canal treatment should be performed even if the tooth responds positively to the pulp sensibility test.
3	Root canal treatment is the first treatment option to consider in the presence of a complicated crown fracture in permanent teeth with incomplete root development.
4	An injured tooth with clinical signs of root fracture can appear normal in conventional radiographs. If suspected, the tooth should be further evaluated with advanced imaging modalities such as cone-beam CT.
5	The splinting duration should be extended in root fractures close to the cervical region.
6	Root canal treatment should be performed on the teeth in the affected segment in an alveolar fracture, even if there are no other injuries to the teeth.
7	In the presence of a tooth fragment in an uncomplicated crown fracture involving dentin, the tooth fragment should be rehydrated in water or saline solution for 20 minutes before it is bonded back to the injured tooth.
8	4-week splinting is required in an alveolar fracture to stabilize the mobile segment.
9	Apart from repositioning displaced teeth, the movable bone segment in alveolar bone fractures can be held in place using teeth splinting.
10	When partial pulpotomy is indicated, non-setting calcium hydroxide or non-staining calcium silicate cements are recommended materials for pulp wound capping.
11	Radiographs of the lips or cheeks should be taken when there are soft tissue injuries and missing fragments of fractured teeth.
12	No endodontic treatment should be initiated at the first visit for root fractures. Instead, the tooth should be monitored for healing of the fracture as well as pulp status.
13	An occlusal radiograph is not usually recommended in crown fracture cases limited to the cemento-enamel junction.
14	An alveolar fracture should be suspected when several teeth in the same segment are moving together on examination.
15	Tenderness to percussion is a common clinical finding when the fracture of a tooth extends to the root surface.
16	Radiographs taken with different angulations should be prescribed by the dentists if fractures involve the root.
17	In an alveolar fracture, teeth in the fractured segment may not respond to the pulp sensibility test. However, root canal treatment should not be initiated at the first visit since false negative results are common for several months after injuries.
18	The apical part of a fractured root usually undergoes necrosis, leading to the need for root canal treatment.
19	Root canal treatment is the treatment of choice when a fractured mature tooth exhibits extensive loss of tooth structure, and the post placement is needed to create retention of the prosthesis.
20	Typically, enamel infraction does not need any treatment. However, etching and sealing with resin is applicable in cases of severe infraction.
21	A calcium hydroxide lining should be placed only when there is a small, visible bleeding from the pulp wound.
22	To achieve physiologic stabilization in root fracture cases, the stainless steel wire applied with composite resin to create a wire-composite splint needs to be larger than 0.4 mm in diameter.
23	At the initial visit of root-fractured teeth, root canal treatment should not be initiated since there is a possibility that damages to neural components of the pulp could result in a negative response to pulp sensibility testing.
24	Enamel-dentin fracture with pulp exposure needs follow-up visits at 6 to 8 weeks, 3 months, 6 months, and 1 year after injury, while the same injury without pulp exposure does not require follow-up visits at 3 months and 6 months after injury.
25	There is a high risk of post-traumatic injury complications in fractured teeth; external inflammatory resorption and pulp canal obliteration should be taken into account while developing a treatment plan.

(*Continued*)

 © 2025. The Author(s).

Supplementary Table S2 (*Continued*)

General information	
Luxations	
1	When a corticosteroid/antibiotic paste is used as an intracanal medicament for severely extruded teeth, it should be placed 1 to 2 weeks after trauma and left in situ for at least 6 weeks.
2	Positive responses to percussion and palpation tests are usually observed in subluxated teeth.
3	At the follow-up visit of the patient with a history of concussion, dentists should start root canal treatment as soon as possible if the affected tooth does not respond to the pulp sensibility test.
4	Abnormal tooth mobility is the key feature of subluxated teeth, while tooth displacement is usually observed but is not always present.
5	Splint duration of an extrusively luxated tooth should be extended when there is evidence of concurrent tooth fractures.
6	A high metallic sound on percussion is a sign of lateral luxation and intrusive luxation.
7	Mature permanent teeth luxated laterally should be monitored closely. Root canal treatment should be started only when there are signs of inflammatory external resorption.
8	Root canal treatment is indicated in mature permanent teeth with lateral luxation to prevent the development of replacement resorption.
9	The laterally luxated tooth should be repositioned digitally and splinted to adjacent teeth for 4 weeks.
10	At the emergency visit, the intruded immature permanent teeth should be allowed to reposition spontaneously instead of using orthodontic repositioning.
11	Orthodontic repositioning is recommended for intruded immature permanent teeth that are not able to re-erupt within 4 weeks.
12	For subluxation, extrusive luxation, lateral luxation, and intrusive luxation, a follow-up visit must be appointed at 2 weeks after injury.
13	The affected teeth in extrusive, lateral, and intrusive luxations are likely to have no response to pulp sensibility tests for several months.
14	Horizontal shift tube technique and occlusal radiography could help visualize a widened periodontal ligament space in lateral luxation cases.
15	Bleeding from gingival sulcus may be present in subluxation.
16	One of the unfavorable outcomes of luxation injuries is that the affected teeth do not respond to the pulp sensibility test within a few months after injuries.
17	The involved tooth should always be splinted to adjacent teeth in subluxation.
18	Root canal treatment should be performed immediately in subluxation cases without any other injuries.
19	The elapsed time is important in the repositioning of an extruded permanent tooth.
20	It should be considered that there might be an accompanying alveolar bone fracture in every lateral luxation case.
21	Either surgical or orthodontic repositioning is necessary for mature teeth intruded between 3 and 7 mm.
22	Mature permanent teeth intruded more than 7 mm should be repositioned orthodontically.
23	The key difference between concussion and subluxation is pulp status or vitality.
24	The extruded tooth should be repositioned and splinted to adjacent teeth for 2 weeks.
25	Occurrence of pulp canal obliteration after severe luxation injuries indicates necrosis of pulp tissues. Therefore, root canal treatment should be attempted to prevent infection or inflammation in the periapical region.
Avulsion	
1	Root canal treatment should be started in an avulsed mature permanent tooth at day 10 to 14 post-replantation.
2	The storage condition and extraoral time of an avulsed tooth affect the periodontal ligament cell survival.
3	Tetanus vaccine status must be inquired about in every patient with an avulsion.
4	Avulsion is the injury type with the highest risk of ankylosis.
5	Avulsed teeth replanted immediately at the site of the accident are more likely to have favorable treatment outcomes compared with other scenarios.
6	In cases of permanent tooth avulsion with more than 60 minutes extraoral dry time, replantation is still recommended.
7	Before the replantation of an avulsed tooth, the tooth should be rinsed with saline solution to remove any debris. Furthermore, it is recommended to soak the tooth in topical antibiotics for 20 minutes to enhance revascularization.

 © 2025. The Author(s).

Supplementary Table S2 (*Continued*)

General information	
8	Dentists should be aware of avulsed tooth storage; the tooth should not be left air-dry while they are taking the history and examining the patient.
9	Recommended storage media for an avulsed tooth are milk, Hanks' Balanced Salt Solution, saliva, or saline.
10	Instead of using water as a storage medium for an avulsed tooth, it is better to leave the avulsed tooth to air-dry.
11	Saliva is preferred to milk as a storage medium for an avulsed tooth.
12	Periodontal ligament cells are likely to be non-viable if the time out of the mouth is more than 60 minutes. However, survival of PDL cells should be expected in the same situation if it is placed in an appropriate medium.
13	Local anesthetics with vasoconstrictors are preferred for the management of avulsed teeth.
14	Pulp revascularization is the goal of immature permanent teeth replantation.
15	Delayed replantation of a permanent tooth is expected to result in replacement root resorption.
16	The correct position of a replanted tooth should be verified not only clinically but also radiographically.
17	Without any other injuries, an avulsed tooth is usually splinted with adjacent teeth for only 2 weeks.
18	Prolonged splint duration may be indicated when the avulsed teeth have not completed their root development yet.
19	Dentists should advise the use of a 0.12% Chlorhexidine gluconate mouthrinse for 2 weeks after tooth replantation.
20	Periodontal healing should be recorded at the follow-up visit after avulsion of permanent teeth.
21	Follow-up visits should be more frequent in replanted immature permanent teeth compared with mature permanent teeth due to the risk of inflammatory root resorption.
22	Pulp canal obliteration is considered a failure of immature permanent teeth replantation.
23	Nylon fishing line can be alternatively used to splint an avulsed tooth to adjacent teeth, especially in pediatric patients having few permanent teeth.
24	Use of systemic antibiotics after replantation of an avulsed tooth is recommended.
25	Doxycycline should be prescribed as the first choice of systemic antibiotics after avulsion due to its antimicrobial, anti-inflammatory, and anti-resorptive effects.
TDIs in the primary dentition	
1	All traumatic dental injuries to the primary dentition require the first follow-up visit at 1 week after the accident.
2	Periodontal healing should be recorded at the follow-up visit after avulsion of primary teeth.
3	In young children, application of 0.12% chlorhexidine gluconate using a cotton swab after a traumatic dental injury is preferred to rinsing.
4	In addition to regular follow-up regimens, a child experiencing an alveolar fracture, intrusive luxation, or avulsion should be evaluated clinically again at 6 years old.
5	Possible disturbances of developing permanent teeth, such as tooth malformation, impaction, and altered eruption, should be informed to the parents after traumatic dental injuries in children.
6	Splinting may be needed in root fractures, alveolar fractures, and lateral luxations of primary dentitions.
7	Pulpectomy is usually indicated in primary teeth with a complicated crown fracture.
8	Treatment options for root fractures in primary dentition range from no treatment to extraction or splinting, depending mainly on the mobility and position of the coronal fragment of the affected tooth.
9	Dentists must always perform a radiographic examination at the follow-up visit of a crown fracture involving dentin in primary teeth due to its high risk of pulp pathosis.
10	It is recommended that intruded primary teeth should be allowed to reposition spontaneously, independent of the degree of intrusion.
11	Extruded primary teeth should be repositioned and splinted by the dentists under local anesthesia at the emergency visit. After reposition, a follow-up visit must be set up in 1 week for splint removal.
12	Transient discoloration of traumatized primary teeth is possible and considered a favorable outcome.
13	Instead of a composite resin, a non-setting calcium hydroxide paste should be covered on exposed dentin of primary teeth with crown fractures involving dentin before placing a restoration to protect the pulp from undesirable stimuli.
14	At the initial visit, the laterally luxated primary tooth should be evaluated radiographically using a periapical radiograph or an occlusal radiograph. Apical periodontal ligament space widening can be observed in the occlusal radiograph if the tooth is displaced labially.

(*Continued*)

 © 2025. The Author(s).

Supplementary Table S2 (*Continued*)

General information	
15	Class IV preparation followed by placing proper restoration is suggested in fractured primary teeth involving enamel alone to restore both function and aesthetics.
16	Cleaning of the injured area using a cotton swab and alcohol-free 0.12% Chlorhexidine gluconate should be advised twice a day for one week after traumatic dental injuries of primary dentition.
17	Reposition of the mobile segment and 4-week splinting should be performed in alveolar fractures affecting primary teeth.
18	After avulsion of a primary tooth, radiographic investigations may be needed to confirm that there are no intruded fragments in the socket.
19	An avulsed deciduous tooth should be replanted.
20	Child abuse should be suspected if the history of the accident does not match or is not consistent with the clinical findings in traumatic dental injuries in young patients.
21	A pulp sensibility test should be performed to evaluate pulp status in traumatized primary teeth.
22	When a diagnosis of root fracture is obtained in deciduous teeth and the crown is excessively mobile and displaced, dentists can either remove the coronal fragment of the tooth and leave the apical fragment in place to be resorbed or reposition and splint the coronal fragment of the tooth for 4 weeks, depending on the child's ability to tolerate dental procedures.
23	Extraction should be considered in cases of lateral luxation in deciduous teeth with a risk of tooth aspiration.
24	Deciduous teeth with luxation injuries usually undergo spontaneous reposition in 6 months. However, the tooth should be repositioned using orthodontic aids in case of severe extrusive luxation.
25	Yearly follow-up until eruption of permanent teeth is needed for monitoring deciduous teeth with a history of lateral luxation or root fracture.

Supplementary Table S3 Mean accuracy and standard deviation of each traumatic dental injury category

		95% confidence interval		SD	*p*-Value
**Types of injury**	**Mean[a]**	**Lower limit**	**Upper limit**		
**General introduction**	**85.26[A]**	82.70	87.81	7.44	<0.0001
**Fractures**	**85.14[A]**	83.56	86.73	4.61	
**Luxations**	**68.23[B]**	65.61	70.85	7.64	
**Avulsion**	**75.20[B,C]**	72.82	77.58	6.92	
**TDIs in the primary dentition**	**75.43[C]**	73.57	77.29	5.41	

Abbreviations: SD, standard deviation; TDI, traumatic dental injury.

[a]Different superscript letters (A, B, and C) indicate statistically significant differences at 0.05 significance level ($p<0.05$).

 © 2025. The Author(s).
